# DEAH-RHA helicase•Znf cofactor systems in kinetoplastid RNA editing and evolutionarily distant RNA processes

**DOI:** 10.14800/rd.1336

**Published:** 2016-06-06

**Authors:** Jorge Cruz-Reyes, Blaine H.M. Mooers, Zakaria Abu-Adas, Vikas Kumar, Shelly Gulati

**Affiliations:** 1 Department of Biochemistry and Biophysics, Texas A&M University, College Station, TX 77843, USA; 2 Department of Biochemistry & Molecular Biology, University of Oklahoma Health Sciences Center, Oklahoma City, OK 73104, USA

**Keywords:** DEAH-RHA RNA helicase, Zinc finger protein cofactor, REH2, MOG helicase, DXH30, Antivirus ZAP, MOP-I, ^H2^F1, Kinetoplastid RNA editing, Trypanosoma brucei mitochondria

## Abstract

Multi-zinc finger proteins are an emerging class of cofactors in DEAH-RHA RNA helicases across highly divergent eukaryotic lineages. DEAH-RHA helicase•zinc finger cofactor partnerships predate the split of kinetoplastid protozoa, which include several human pathogens, from other eukaryotic lineages 100–400 Ma. Despite a long evolutionary history, the prototypical DEAH-RHA domains remain highly conserved. This short review focuses on a recently identified DEAH-RHA helicase•zinc finger cofactor system in kinetoplastid RNA editing, and its potential functional parallels with analogous systems in embryogenesis control in nematodes and antivirus protection in humans.

## Introduction

RNA and their ribonucleoprotein complexes (RNPs) in nearly all aspects of gene expression are remodeled by helicases, which are nucleoside triphosphate dependent molecular motors that unwind double helical nucleic acids ^[1–4]^. All helicases are divided into six superfamilies (SFs) based on amino acid sequence and structure. The eukaryotic RNA helicases are monomeric and belong to SF1 and SF2. The ring-forming helicases (typically hexameric) belong to SF3-to-6. SF1 and SF2 helicases are further divided into several protein families and subfamilies. The DEAH-RHA subfamily helicases are placed within SF2 and are found in all eukaryotes. These proteins are named after the sequence Asp-Glu-Ala-His (DEAH), which is also known as the Walker B motif or motif II, and after the RNA helicase A (RHA), which is also a member of SF2. Several excellent recent reviews have described in detail the function and architecture of RNA helicases in general and DEAH-RHA RNA helicases in particular ^[1–4]^.

Highlighting the wide distribution of DEAH-RHA helicase-dependent processes is the participation of these proteins in a unique process of RNA editing in the single mitochondrion of kinetoplastid protozoa ^[5–7]^. This processing involves site-specific insertion and deletion of uridylates at many mRNA sites in protein-catalyzed reactions directed by small guide RNAs (gRNAs). The gRNAs exhibit complementarity to fully edited mRNA through canonical and G•U base pairs. In *Trypanosoma brucei*, the causative agent of human African trypanosomiasis or sleeping sickness ^[8]^, the holo-editosome targets most mitochondrial mRNAs and consists of over 30 proteins, including two RNA helicases, one of which is a DEAH-RHA helicase ^[5, 7, 9–11]^. Ribosome biogenesis in yeast and humans requires the largest number of different helicases, including several DEAH-RHA proteins. The spliceosome, which comprises over a hundred proteins, also requires a large set of RNA helicases, including four different DEAH-RHA proteins in yeast ^[1, 2, 12, 13]^.

DEAH-RHA helicases participate in almost every kind of RNA processing reaction in RNA biology and are specifically regulated by a class of protein cofactors that typically contain G-patch domains. The characterized G-patch protein cofactors have been reviewed in detail ^[14]^. The single DEAH-RHA helicase in kinetoplastid RNA editing does not have a typical G-patch protein cofactor, but instead it binds a zinc finger (Znf) protein cofactor. Only two other DEAH-RHA helicase•Znf cofactor systems have been reported so far. One system participates in the control of embryogenesis in nematodes and the other system participates in antivirus protection in humans ^[15, 16]^. The presence of a DEAH-RHA helicase•Znf cofactor partnership in trypanosomes, nematodes, and humans suggests that this partnership is widespread in eukaryotes.

This review compares the sole DEAH-RHA helicase in kinetoplastid RNA editing with other subfamily members and examines the available studies and functional models for the known DEAH-RHA helicase•Znf cofactor systems. It is feasible that DEAH-RHA helicase•Znf cofactor partnerships share mechanistic features despite their taxonomic distance and their participation in very different RNA processes.

## Domain organization of DEAH-RHA helicases in kinetoplastid protozoa and highly divergent organisms

**Figure 1:**
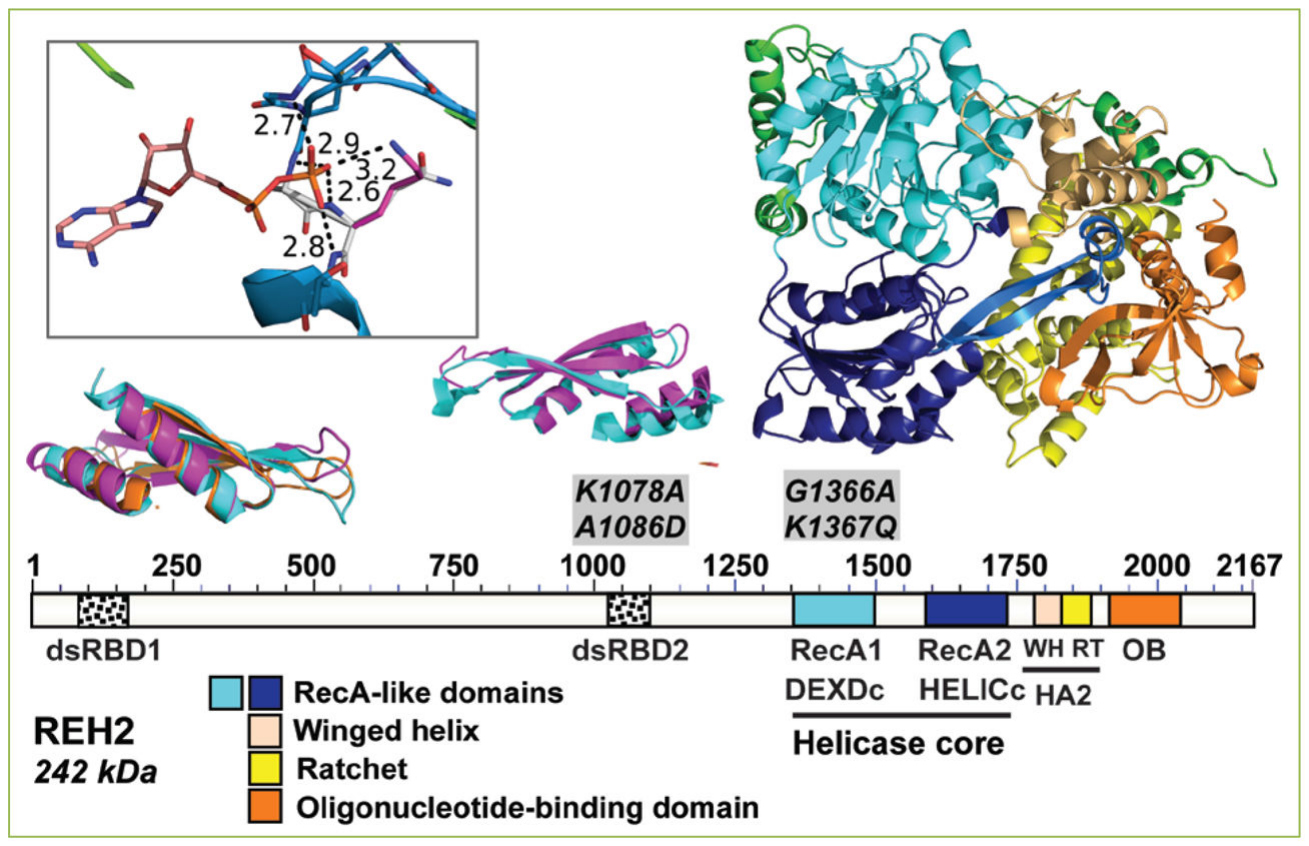
Domain organization of REH2 in *T. brucei* The domain map at scale and the structure model of REH2 use the same color codes. REH2 conserved features in a homology model made using ADP-bound Prp43p (PDB ID:2XAU) as a template. The conserved features include tandem RecA-like domains (DEXDc and HELICc in current domain databases) in the helicase core that are common to SF1 and SF2 helicase superfamilies. DEAH-RHA subfamily helicases have a unique C-terminal cluster of small domains that includes a winged helix and ratchet (together annotated as ‘helicase-associated domain’ HA2 in domain databases) and an OB-fold domain. REH2-specific sequence or elements are depicted in green. REH2 has two predicted dsRBDs: dsRBD1 and dsRDB2. The dsRBD1 is only visible in a structure-based search with Phyre2 ^[47]^. The dsRBD2 is visible in both a sequence-based analysis with the NCBI’s interface CD-search ^[48]^ and the structure search. In the models of dsRBD1 and dsRBD2 in REH2, the Tb protein is in magenta and orange while the archetype dsRBD structure [PDB ID:1DI2, ^[49]^] is shown in cyan. The structures were superposed with Theseus ^[50]^ and had a maximum likelihood rmsd of 1.98 Å for the alpha carbon atoms. Reported K1078A•A1086D mutated sites in dsRBD2 and G1365A•K1366Q in motif I are indicated ^[9, 19]^. The inset shows a homology model of motif I mutations described in ^[19]^. The mutated sites G1365A•K1366Q are shown with the carbons colored white. These mutations in the P loop or motif I (atoms of motif I are shown as sticks) remove one H-bond between the beta phosphate of the ADP and REH2. Four H-bonds remain after the mutations. The molecular model suggests the loss of one H-bond and the positive charge of the Lysine sidechain that should be important in countering the negative charge of the phosphate even though the lysine side chain and the phosphate are widely separated in the model. The molecular models were rendered by PyMOL (The PyMOL Molecular Graphics System, Version 1.8 Schrödinger, LLC).

**Figure 2:**
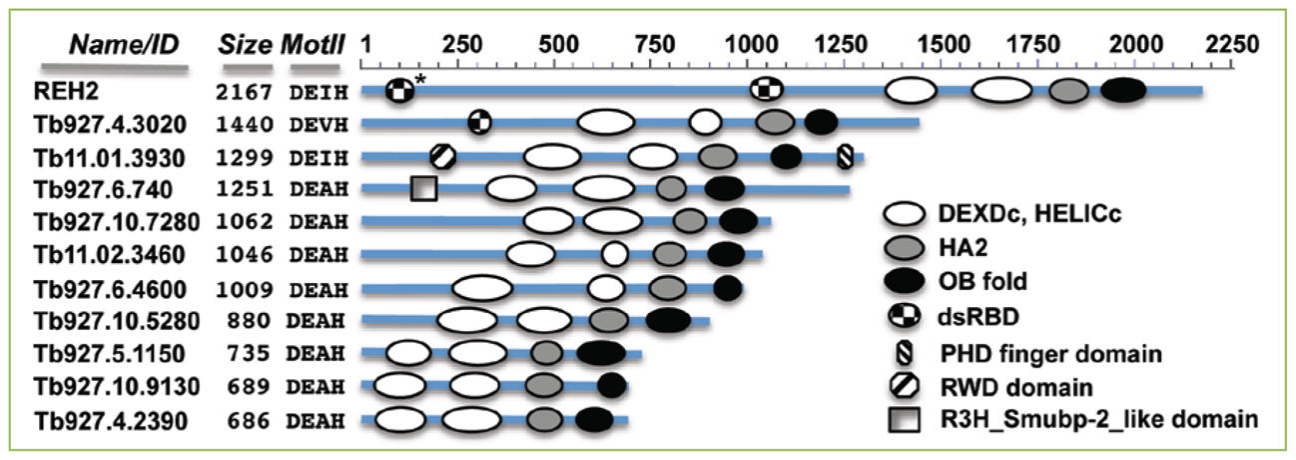
Graphical representation at scale of DEAH-RHA proteins in *T. brucei* Domain annotations were performed using NCBI’s CD-search analyses. In REH2, the dsRBD2 is only visible in a structure search (*) as described in Fig. 1. The REH2 paralog Tb927.4.3020 has a dsRBD proximal to the helicase domain. A second dsRBD is not visible in this protein in either a CD-search or in a Phyre2 search. Functional studies are currently reported only for REH2. Conserved domains besides the DEAH-RHA defining features described in Fig. 1 are: PHD finger (cd15489), RWD domain (cI02687) and R3H_Smubp-2_like domain (cd02641). Also indicated are the number of amino acids for each protein and the DEAH family defining residues located at the motif II of the DEXDc domain (RecA1).

**Figure 3:**
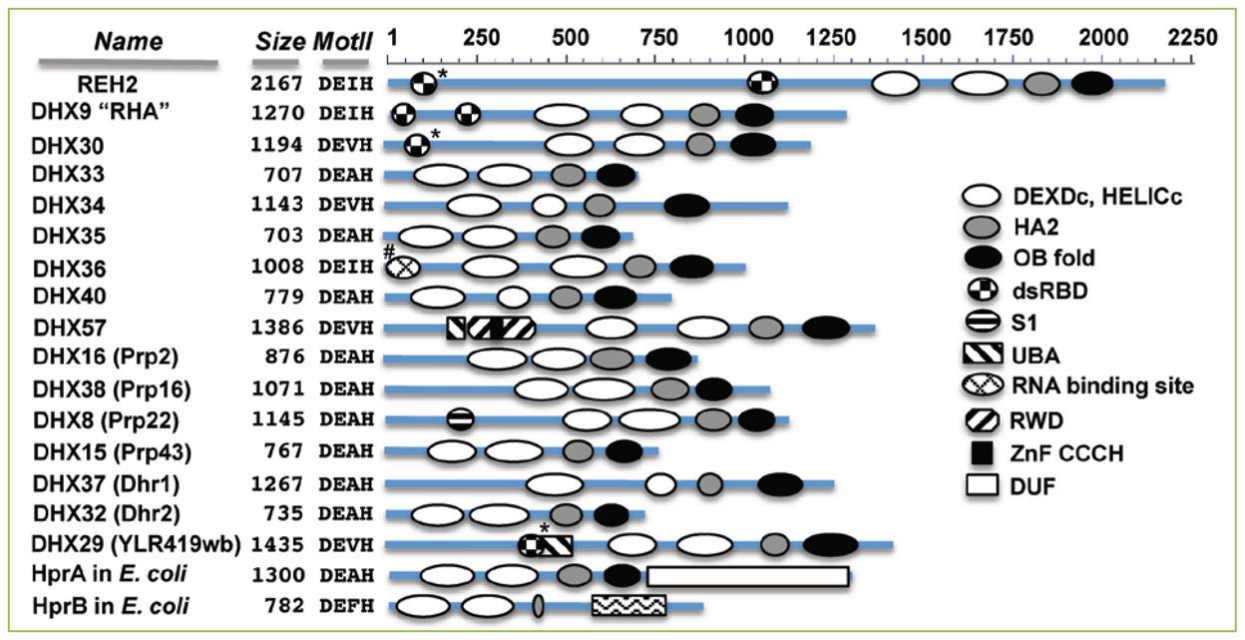
DEAH-RHA proteins in highly divergent lineages The graphical representations are at scale. Predictions from NCBI’s CD-searches gave the domain annotations in the human genes (DHX prefix), in their yeast homologs (in brackets), and in *E. coli* Hpr genes. REH2 was described above. Another study identified the N-terminal dsRBD elements in DHX29 in a structure search (*) ^[51]^. DHX30 has an N-terminal dsRBD annotated in UniProtKB ^[52]^ that was detected with the Phyre2 server but not with a CD-search analysis. An unidentified N-terminal RNA-binding domain in DHX36 was determined experimentally (#) ^[22]^. Other conserved domains that flank the DEAH-RHA defining features are: RWD (pfam05773), UBA_DHX57 (cd14317), UBA_YLR419W_like (cd14271), ZnF_C3H1 (smart05773), S1_DHX8_helicase (cd05684), and DUF3418 (pfam11898). Protein sizes and the DEAH family defining residues in mot II are indicated as in Fig. 2. Some of the proteins listed here have additional conserved domains or sequence features that were not detected in the CD-searches but that have been identified through functional or sequence analyses. For example, RHA has a domain for binding to RNA polymerase II between dsRBD2 and RecA1. Also, the C-terminus of RHA includes two RGG-boxes and nuclear localization/export signals ^[24]^.

**Figure 4:**
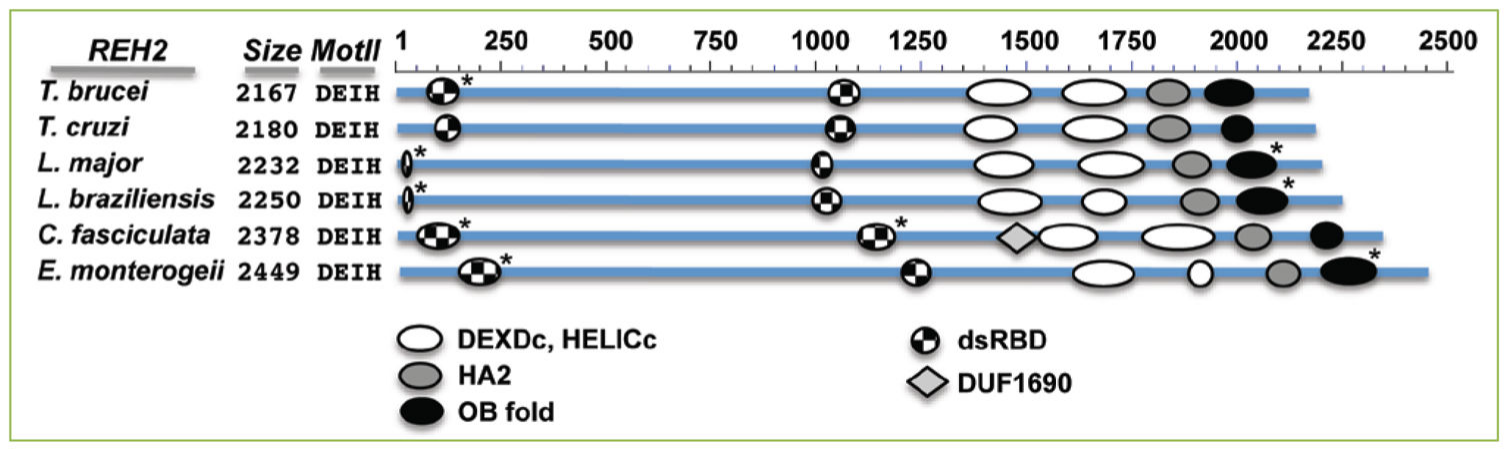
Graphical representation at scale of REH2 orthologs in kinetoplastids Domain hits were identified in NCBI’s interface CD-searches ^[48]^. The CD-search detected a N-terminal dsRBD only in the *T. cruzi* protein, and an OB-fold in some of the proteins. Three-dimensional structure predictions (*) from the Phyre2 server ^[47]^ identified a N-terminal dsRBD and a OB-fold with high confidence in all orthologs. In the *Leishmania* orthologs, only part of a typical dsRBD was detected. The IDs and sequences of the examined proteins were obtained from the kinetoplastid genome resource TriTrypDB ^[53]^. The species and ID are given as follows: *T. cruzi* (TcCLB.511003.30 or XP_8160321), *L. major (*LmjF.34.3230), *L. braziliensis (*XP_001564561), *C. fasciculata (*CFAC1_290061800 ) and *E. monterogeii (*EMOLV88_340032900). Also indicated are the number of amino acids in each protein and the DEAH family defining residues in motif II.

SF1 and SF2 helicases have a catalytic core of tandem RecA-like domains with typical motifs for ATP-binding and hydrolysis and for RNA binding and unwinding. The first crystal structure of a DEAH-RHA protein revealed a cluster of domains at the C-terminus that distinguishes it from other members of the DEAH family ^[17]^. This cluster includes a winged helix motif, a ratchet motif, and an oligonucleotide-binding (OB-fold) domain. Some DEAH-RHA proteins have additional domains on the N-terminal side of the catalytic core of the helicase. These N-terminal domains are thought to be crucial for facilitating the recruitment of specific cofactors and the RNA target, and for modulating the function of the helicase activity. The kinetoplastid RNA helicase termed REH2 (RNA Editing Helicase 2) has all of the conserved features of DEAH-RHA proteins (Fig 1) ^[10]^. Sequence analyses of helicase core motifs in all SF1 and SF2 helicases in trypanosomes identified 13 DEAH family members ^[18]^. Further sequence and structural predictions indicate that all of these proteins are also RHA subfamily members (Fig. 2), except for Tb09.211.4430 and Tb927.4.3890. REH2 is the largest DEAH-RHA helicase and the only family member characterized in kinetoplastids ^[9, 10, 19–21]^. However, a comparison of the orthologs in different kinetoplastids, including *T. brucei, T. cruzi* and *Leishmania*, revealed a DEAH family member that is unique to *T. cruzi* and another member that is present in trypanosomes but not in *Leishmania* ^[18]^. REH2 is also substantially larger than the characterized DEAH-RHA proteins in humans, yeast, and bacteria (Fig. 3). REH2 has two dsRNA-binding domains (dsRBDs). The first (dsRBD1) is near the N-terminus of the protein, and the second (dsRBD2) is near the N-terminus of the catalytic core. This domain organization is common in REH2 orthologs in kinetoplastids (Fig. 4). REH2 has a shorter paralog gene (Tb927.4.3020) ^[9, 11]^ that carries only one N-terminal dsRBD domain. The possible function(s) of Tb927.4.3020 remains undetermined; initial studies using RNAi-inducible down-regulation were unable to link this protein to RNA editing ^[19]^. Only the RHA helicase in humans (DHX9) is known to carry two dsRBDs, while DHX30 and DHX29 each carry a single predicted dsRBD. Other RNA binding sites have been experimentally determined (DHX36) ^[22]^ or predicted (DXH8) ^[23]^ (Fig. 3). It is possible that other auxiliary domains in these proteins participate in nucleic acid binding. The complex modular arrangement that defines the DEAH-RHA helicase subfamily evidently appeared early in evolution because it is present in bacteria and viruses ^[1, 2]^. Because the DEAH-RHA modular structure is well conserved in biology, members of this subfamily are distinguished from each other by different sets of auxiliary domains mostly found at their N-terminus. A variety of specialized auxiliary domains often provide important functional information. Consistent with the early split of kinetoplastids from other eukaryotes, the sequences outside the DEAH-RHA domains have no obvious homology to yeast or human proteins. The assignment of orthologs in kinetoplastids to specific roles and processes will require detailed functional studies.

The N-terminus of both kinetoplastid REH2 and human RHA (DHX9) helicases carry two dsRBDs; however, the distance in sequence that separates the dsRBDs is very different between the two proteins (Fig 3). The presence of the two dsRBDs suggests that REH2 and RHA have mechanistic similarities, but it does not imply a shared role in related processes. RHA participates in different cellular processes with implications in human diseases including several cancers and viral infections. These include control of DNA replication, transcription, translation, microRNA biogenesis, RNA processing and transport, genomic stability, and retroviral gene expression ^[3, 14, 24]^. REH2 participates in RNA editing in kinetoplastid mitochondria. Additional roles for REH2 are feasible but not established yet. An early study of the REH2-associated RNP speculated that this complex serves as an “organizer” of mitochondrial genome expression ^[9]^. Indeed, isolations of REH2 are enriched in mitoribosomes and other mitochondrial complexes known to participate in mRNA 3′ maturation and stability ^[5, 9]^. Among the DEAH-RHA proteins in kinetoplastids, a few additional putative auxiliary domains with relatively low E-values can be detected. These domains include a RWD domain (named after three major RWD-containing proteins: RING finger and WD repeat containing proteins, and DEXDc-like helicases) ^[25]^ in Tb11.01.3930 that is also observed in the human DHX57 protein (Figs. 2 and 4). As mentioned above, the annotation of auxiliary domains is insufficient to enable confident predictions of their functional roles. Yet, this information hints to possible mechanistic similarities in the way these proteins may operate.

## Control of editosome assembly in the single mitochondrion of kinetoplastid protozoa

**Figure 5:**
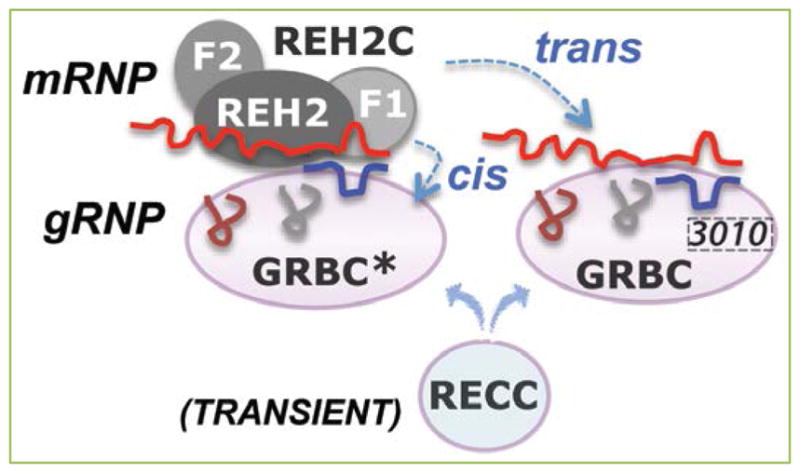
Holo-editosomes include assemblies of auxiliary RNPs and the editing enzyme This review focuses on the REH2C subcomplex (with its protein subunits indicated as gray ovals). This subcomplex includes the REH2 helicase, two cofactors (^H2^F1 and ^H2^F2), and all mRNA types involved in editing (pre-edited, partially edited intermediates, and edited). GRBC* and GRBC are variants of another subcomplex that contains gRNAs and several proteins. These gRNA-bound variants (gRNPs) are distinguished by their content of a protein subunit (3010). REH2C binds to GRBC* via stable contacts (*in cis*) and to GRBC via transient contacts (*in trans*). Both types of interaction are via RNA. ^H2^F1 is proposed to recruit mRNA targets for REH2-catalyzed unwinding of localized secondary structure. The relatively disentangled RNA conformers are more likely to anneal with gRNA or undergo editing at individual sites. The preassembled mRNA-gRNA hybrids in the RNP scaffolds can then be processed by the RECC editing enzyme. Thus, the REH2•^H2^F1 system modulates RNA hybrid quality in assembled RNP scaffolds and the ensuing addition of RECC enzyme in complete holo-editosomes. The initiating gRNA (colored blue) hybridizes to the 3′ most block in the pre-edited mRNA. The mRNAs are shown in red, and the gRNA transcripts are shown in various colors.

**Figure 6:**
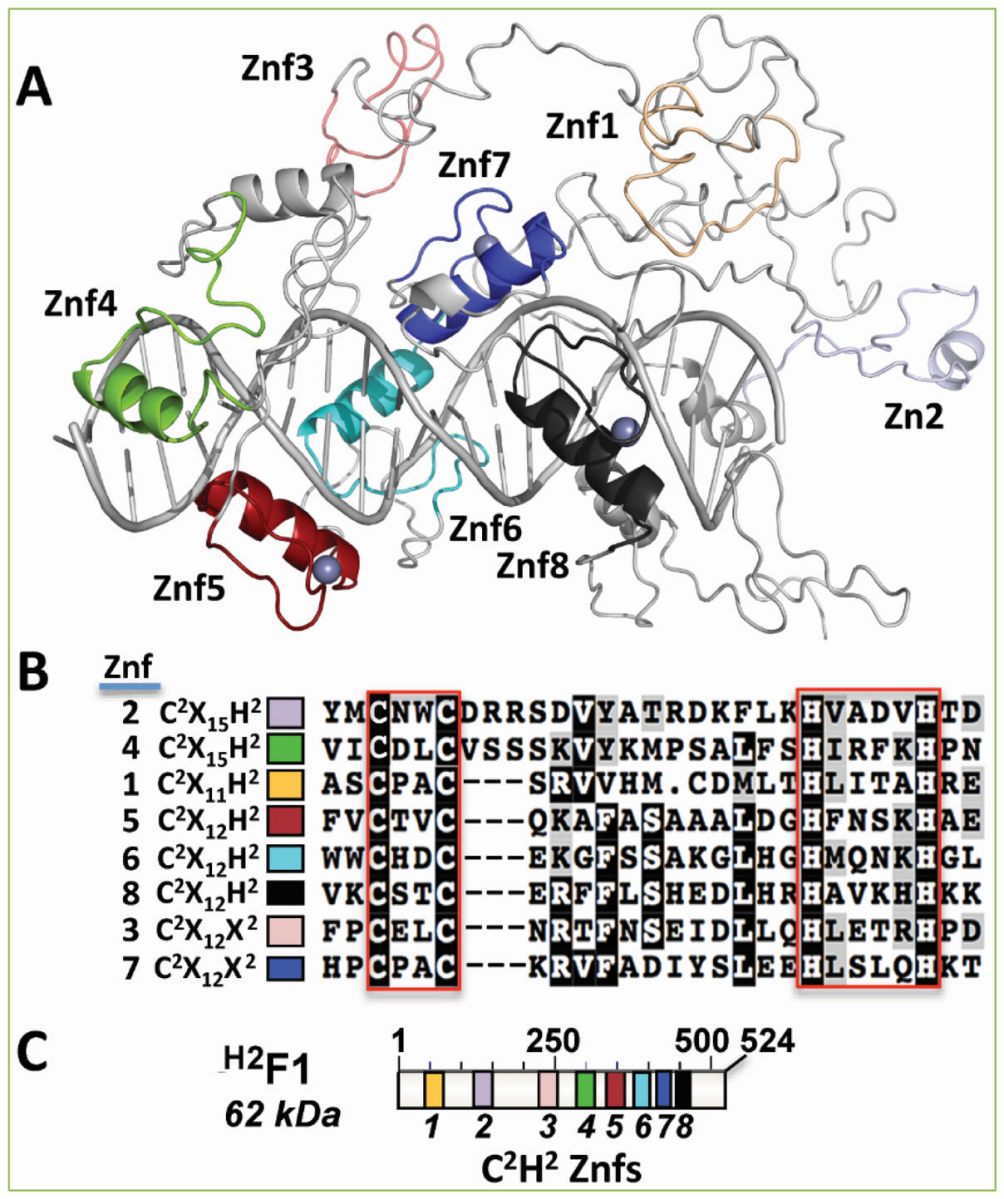
Domain organization of ^H2^F1 (A) Structure model of ^H2^F1 with the location of 8 potential zinc fingers (Znf1-8). Znf1-4 were found by visual inspection. Znf5-8 appeared in conserved domain databases. Znf5 is predicted to bind double-stranded RNA. The position of the zinc ligands was precise enough for the placement of the zinc atoms for three of the zinc fingers (gray balls by Znf5, Znf7, and Znf8). A DNA ligand was proposed by structural similarity with DNA-bound Aart (PDB ID:2i13), a designed six-finger zinc finger protein ^[54]. H2^F2 has a glycine-rich C-terminus. (B) Shaded multiple-sequence alignment of all 8 putative zinc fingers in ^H2^F1. The two conserved cysteines and two conserved histidines that are predicted to co-ordinate a zinc ion are enclosed by the red boxes. (C) Graphical representation of ^H2^F1 at scale. Znf1-8 are color coded as in panels A and B.

The holo-editosome in *T. brucei* includes the multi-subunit RECC (RNA editing core complex) enzyme and a large number of auxiliary proteins. Most auxiliary proteins are found in ribonucleoprotein subcomplexes with mRNA and gRNA ^[10, 19, 20, 26]^. One of these RNPs, the REH2-associated protein subcomplex (REH2C) (Fig. 5) includes the REH2 helicase, two cofactors, and all mRNA classes that participate in editing (i.e., pre-mRNA substrates, partially edited intermediates, and fully edited products) ^[10, 19, 20]^. This mRNA-associated ribonucleoprotein subcomplex (mRNP) associates with variants of a gRNA-associated RNP (gRNP) (Fig 5). The mRNP binds the gRNP variants via either stable or transient RNA-mediated contacts ^[10, 19]^. A photo-crosslinking experiment with a model mRNA-gRNA hybrid substrate identified a close interaction (≤4 Å) of REH2 with the editing site of the substrate ^[9, 19]^. A model of editosome assembly proposes that mRNP basepairing with gRNPs leads to the formation of mRNA-gRNA hybrid substrates. The first editing site (ES1) in the mRNA is the position just 5′ of a short initial duplex between mRNA and gRNA (termed the anchor duplex) ^[27, 28]^. The ES2 is established as the anchor duplex incorporates correctly edited ES1 sequence. This cycle is repeated one site at the time as the editing machinery advances along the mRNA in a 3′-to-5′ direction. Transient addition of the RECC enzyme to preassembled substrate-loaded multi-RNP scaffolds would establish higher-order catalytic holo-editosomes ^[10]^. REH2 and one of its cofactors (^H2^F1) with eight predicted zinc fingers are required for efficient editing *in vivo* (Fig. 6). This was shown by RNAi-mediated silencing experiments ^[10, 20, 21, 29]^. The REH2C subcomplex carries an ATP-requiring 3′-5′ unwinding activity that is linked to the REH2 helicase ^[9]^. In purifications of REH2 from mitochondrial extract, the unwinding activity is inhibited by mutation of conserved carboxylates in the catalytic motif I (in RecA1) or in dsRBD2 (Fig. 1). Mutations in either domain also prevent copurification of REH2 with mRNA and gRNA ^[9, 19]^. In agreement with the RNP docking model, the loss of mRNA association leads to a collapse of the stable mRNP-gRNP assembly ^[19]^. Another prediction of the model is that mRNP copurification with the RECC editing enzyme requires gRNA (presumably most or all gRNA molecules are part of gRNPs). Indeed, a loss of mRNP-RECC association is observed upon depletion of gRNA in mitochondria ^[10]^. Thus, formation of complete multi-RNP scaffolds enables the transient association of the RECC enzyme with the helicase mRNP. This finding also supports the concept of RECC transient addition through its binding to preassembled mRNA-gRNA duplexes in the multi-RNP scaffolds ^[10]^. A ^H2^F1 knockdown prevents the normal association of the REH2 helicase with gRNPs and the RECC enzyme. ^H2^F1 is a proposed regulator of REH2 that controls both the docking of this helicase with gRNPs and the addition of the RECC enzyme to holo-editosomes ^[10]^. Consistent with the proposed REH2•^H2^F1 direct interaction *in vivo*, recombinant versions of REH2 and ^H2^F1 bind directly with each other to form a stable complex *in vitro* ^[10]^. Yet, additional studies are needed to establish how ^H2^F1 and REH2 act in concert during the assembly of substrate-loaded holo-editosomes.

## Embryogenesis control in *nematodes*

**Figure 7:**
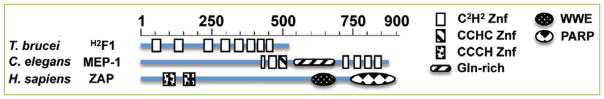
Domain organization at scale of known zinc finger cofactors of DEAH-RHA proteins Domain annotations in NCBI’s CD-search predictions. Identification of the zinc fingers in ^H2^F1 is described in Fig. 6. The domains in MEP1 (Q21502 - MEP1_CAEEL) are not detected in a CD-search analysis but are annotated in UniProtKB. Predicted Znf domains in ZAP isoform 1 (Q7Z2W4 (ZCCHV_HUMAN) are not detected in a CD-search analysis but are annotated in UniProtKB and were identified in a crystal structure of the N-terminal fragment in a homolog protein ^[16]^. Detected conserved domains are WWE (smart00678) and PARP (pfam00644).

*C. elegans* can exist as sequential hermaphrodites. Sperm production occurs during development and oogenesis occurs in adults. The switch from sperm production to oogenesis requires a controlled posttranscriptional repression of *fem-3*, a sex-determining gene that promotes male development ^[30–32]^. The switch from sperm to oocyte production is controlled at different levels including by three critical nuclear DEAH-RHA helicases: MOG-1, MOG-4 and MOG-5 (“MOG” stands for Masculinization Of the Germline) ^[15]^. These proteins are the orthologs of the DEAH-RHA helicases Prp16, Prp2, and Prp22 in yeast. A cofactor of these MOG proteins, MEP-1 (for MOG-interacting and ectopic P-granules) (Fig. 7) is also essential for the sperm⇒oocyte switch. MEP-1 has seven predicted Znf domains (Fig 7). Binding of MEP-1 to each MOG protein was confirmed in yeast two-hybrid assays and in experiments using *in vitro*-translated proteins. Disruption in the expression of MEP-1 or any of its MOG partners prevents the required repression of *fem-3* mRNA at the translation level. This results in the masculinization of the germline (the “mog phenotype”) ^[15]^. Because MOG and MEP-1 are nuclear proteins, it has been unclear how the cytosolic inactivation of *fem-3* mRNA takes place. A MEP-1 homolog of unknown function was identified in Drosophila but not in yeast, suggesting that MEP-1 evolved among metazoans. Recombinant MEP-1 binds RNA non-specifically *in vitro*, although the *fem-3* mRNA is the only known cognate target of MEP-1 ^[15]^. MEP-1 alone or with its helicase partners or other cofactors may provide the required RNA specificity *in vivo*. Prp16, Prp2, Prp22, and their human orthologs control steps of pre-mRNA assembly with several snRNPs. Thus, the MOG•MEP-1 system could control similar steps. Yet, splicing defects were not observed in *mog-1* null mutant animals ^[33]^. It was speculated that the MOG•MEP-1 system mediates epigenetic effects that are coupled with splicing. One such effect is the deposition of the exon junction complex (EJC) near exon-exon junctions of mRNAs. This post-splicing accumulation of EJCs by the spliceosome machinery is known to affect the fate of mRNAs such as nuclear export, degradation, subcellular mRNA localization, and translational yield ^[34]^. Consistent with this idea, two core components of EJC in *C. elegans* are also required for the sperm⇒oocyte switch ^[35, 36]^. The precise mechanism of action of the MOG•MEP-1 system remains to be determined.

## Antiviral response in vertebrates

The zinc-finger antiviral protein (ZAP) is a host factor that inhibits the replication of a broad spectrum of important viruses, including HIV, Ebola virus and Sindbis virus ^[37, 38]^. ZAP has four predicted zinc fingers (Fig. 7), and its activity has been associated with two RNA helicases, the DEAH-RHA protein DXH30 and the DEAD protein p72. In addition, ZAP inhibits human retrotransposons in association with another helicase, MOV10 ^[39, 40]^. ZAP also acts against hepatitis B virus transcription and replication ^[41]^. ZAP specifically inhibits viral replication by a mechanism that involves direct ZAP binding to the viral mRNA and recruitment of the RNA exosome to degrade the viral mRNA target ^[38]^. ZAP activity requires normal expression of the cellular factor DXH30 that carries a conserved N-terminal dsRBD (Fig. 3). ZAP and DXH30 are thought to form a complex *in vivo*. Direct binding between these proteins only requires their N-termini ^[38]^. The ZAP-interacting N terminus fragment in DXH30 includes the dsRBD but not the catalytic core. The DXH30-interacting N terminus fragment in ZAP includes all four zinc fingers. The specific elements or motifs in ZAP and DXH30 that mediate their direct interaction remain to be defined.

The zinc finger motifs of ZAP are required for RNA binding and antiviral activity ^[42]^. The small N terminal fragment of ZAP with all four zinc fingers binds DXH30 and leads to the same antiviral activity as the full-length ZAP. Each of the four zinc fingers in ZAP may be an RNA binding unit because mutation of any of the zinc fingers reduced ZAP’s activity to some extent. ZAP also binds directly with the exosome component hRrp46p through a 30 amino acid binding region. This ZAP-exosome interaction is relevant because depletion of the exosome subunits hRrp41p or hRrp46p significantly reduced ZAP’s antiviral activity ^[38]^. Thus, ZAP is a hub with distinct binding surfaces that bring together the DXH30 helicase, target viral mRNAs, and the RNA processing exosome. It has been proposed that the ZAP antiviral activity involves the removal of secondary structure in bound target mRNAs via DXH30-catalyzed unwinding to facilitate the exosomal nucleolytic degradation of relatively disentangled RNA conformers ^[37]^. The few identified ZAP-responsive RNA fragments have no known sequence motifs. The only common feature in these fragments is that they are at least ~500 nucleotides long, so the source of binding specificity for these RNAs has been elusive. A crystal structure of a N-terminal fragment (residues 1–225) with all four fingers in ZAP provided insights into RNA target recognition by ZAP ^[16]^. The structural features of ZAP (discussed in more detail below) suggest that the target RNA is recognized by its tertiary structure rather than its base sequence. This explains why only a few ZAP-responsive RNAs are known and why they are at least 500 nucleotides long and have no easily identified sequence motifs.

## Do DEAH-RHA helicase•Znf cofactor systems in distant species share common mechanisms of RNA target recognition?

Eukaryotic DEAH-RHA helicase are known to bind a group of regulatory factors termed G-patch proteins, which carry one or more conserved G-patch motifs ^[14]^. G-patch cofactors typically establish direct contacts with the C terminal domains in DEAH-RHA helicases. The G-patch and OB-fold domains act in concert and are thought to provide specificity in RNA binding and activation of RNA-dependent NTPase and unwinding activities.

A second group of DEAH-RHA helicase cofactors are proteins that have multiple zinc-fingers. Three DEAH-RHA helicase•Znf cofactor systems in organisms that diverged 100–400 Ma ^[43]^ were identified in kinetoplastid protozoa, nematodes and humans. All three helicase•Znf protein systems impact the functional or regulatory fate of specialized mRNPs. In trypanosomes, the mitochondrial REH2•^H2^F1 system was proposed to modulate the assembly and remodeling of mRNA-gRNA hybrids that are targeted by the editing enzyme ^[10]^. In the worm germline, the nuclear MOP•MEP-1 system is thought to control epigenetic remodeling of mRNP complexes that modulate the translation fem-3 mRNAs, and thereby, the switch from spermatogenesis to oogenesis. The location of EJCs near exon-exon junctions by the spliceosomal machinery could be used to mark fem-3 transcripts for downstream cytosolic inactivation ^[44]^. In humans, the cytosolic DXH30•ZAP system specifically binds viral mRNA targets and promotes their exosomal degradation ^[37, 38]^. The Znf cofactors modulate the function of their helicase partners in RNA editing, RNA splicing related processes, and the destruction of viral RNAs. It is likely that Znf cofactors can modulate DEAH-RHA helicase in additional RNA processes where they are present.

A critical question that applies to these three helicase•Znf cofactor systems is how they achieve RNA target specificity. The problem is complicated by the fact that multiple related substrates must be recognized with efficiency and specificity. Moreover, substrate specificity may not always involve consecutive sequence-dependent cis-elements. For example, most mitochondrial mRNAs in trypanosomes require editing, and many viral mRNAs are specifically targeted by exosomes. Also, in the lifetime of mRNAs, the mRNPs undergo constant remodeling including the addition and removal of EJC at positions of variable sequence near splice junctions in mRNAs. Thus, neither, the editing substrates, viral mRNAs, or splice junction sites appear to carry sequence-specific motifs that are easy to identify.

The crystal structure of ZAP provides important clues as to how relatively small protein regions with a tandem array of zinc finger domains may achieve complex specificities in RNA recognition ^[16]^. Structural and functional analyses of residues 1–225 in ZAP identified an RNA binding surface involving multiple positively-charged residues. The four Znf motifs in the ZAP structure are positioned to flank two sides of a positively charged cleft that likely binds folded RNA. Model structures of the ZAP-RNA interaction suggested that the target RNA should have a specific tertiary structure to precisely fit into the three-dimensional RNA-binding cleft of ZAP. The same principle may apply to other Znf cofactors, with the level of complexity in RNA recognition influenced by the number in zinc finger domains involved. Thus, protein dimerization, as in ZAP and MEP-1 ^[15, 16]^, would provide a larger platform to coordinate complex RNA features. Moreover, the protein may bind several related RNA targets if each zinc finger contributes differently to the binding of the distinct RNAs. This would meet the need for specific recognition of a large set of diverse editing substrates, viral mRNAs, or splice sites. Thus, in an analogy to ZAP, the tandem zinc finger array in other proteins, including ^H2^F1 and MEP-1, could be arranged to build up specialized modules for the binding of complex RNA tertiary structures rather than specific sequences.

A second critical question is whether or not DEAH-RHA helicase•Znf cofactor systems share a common function. Monomeric DEAH-RHA helicases appear to require a 3′ single-stranded extension, and they are not highly processive. Instead, they promote both local 3′-to-5′ unwinding of discrete structures and RNP assembly ^[1, 2]^. The proposed role of ZAP in removing double-helical structure of 3′ ends in bound mRNAs via DXH30-catalyzed unwinding for exosome-mediated degradation serves as a guide to propose a common function for other zinc-finger cofactors. In kinetoplastid RNA editing, ^H2^F1 could recruit mRNA targets for REH2-catalyzed unwinding of structure that may interfere with annealing of a gRNA next to the editing site. Interestingly, a photo-crosslinking experiment with a synthetic RNA substrate containing a photo-reactive thio-U base detected a close-proximity (≤ 4 Å) contact of REH2 with the editing site. This localized REH2 at the catalytic center of the editosome, and suggests that the helicase could contribute to local unwinding of the substrate including at the editing site, the abutting 3′ residues for gRNA annealing, or both ^[9]^. Finally, MEP-1 may assist in modulating RNA structure of bound sites via MOP-1 catalyzed unwinding. This unwinding may facilitate engagement by spliceosome components including during EJC deposition. In these three systems, the zinc-finger protein alone could specifically bind to the RNA target. Its helicase partner could then catalyze cycles of local structure remodeling on the bound RNA, thus facilitating access and activity of the components of the RNA editing enzyme, exosome, or spliceosome.

Furthermore, a better understanding of the functional synergy between RNA helicases and their cofactors during RNA substrate recognition will involve identification of relevant domains and specific amino acids required for intra-protein and inter-protein contacts in the helicase•cofactor complexes. The requirements for Znf cofactor binding to cognate helicases may exhibit important differences. The N-terminus in both DXH30 and ZAP suffices for association between these proteins *in vivo*. For DXH30, the ZAP-binding region includes a dsRBD but not the catalytic core. In ZAP, the DXH30-binding region has all zinc fingers and renders full virus protection ^[37]^. The direct interaction of MEP-1 with MOG helicases, examined in yeast and with *in vitro*-translated proteins, only required the N-terminus of MEP-1 including its zinc fingers. The MOG helicase requirements for interaction were not examined, so the MEP-1 binding to MOG helicases may involve one or more zinc fingers. Alternatively, determinants outside the zinc fingers may be responsible for the protein-protein interaction. Nonetheless, a recombinant REH2 helicase fragment (residues 1261–2167) and ^H2^F1 form a stable complex *in vitro* ^[10]^. That tested REH2 fragment lacked dsRBD1 and dsRBD2, so the helicase core and the C terminal domain cluster were sufficient to bind ^H2^F1. Only full length ^H2^F1 was tested in that study. Overall, the N-terminus of DXH30 and MOG were shown to bind ZAP and MEP-1, respectively. In contrast, the C-terminus of REH2 sufficed to bind ^H2^F1 *in vitro*. This suggests that these helicases differ in their binding mechanism to their cognate Znf cofactors. An alternative model could be that both N-and C-termini in the RNA helicases engage in direct contacts with their Znf cofactors *in vivo*. Yet, either terminus of the helicase may suffice to detect an association in the applied assays *in vitro* or in yeast.

Structural proteomics of a recombinant complex between Prp43 and its G-patch cofactor Ntr1 mapped primary contacts between the G-patch motif and the helicase C-terminal domains, namely, the winged helix, the ratchet, and the OB-fold domains ^[45]^. However, a few contacts between sites outside the G-patch motif and N-terminus helicase positions were also observed. Potentially, contacts of cofactors with both N- and C-termini of the helicase may lead to higher binding affinities between the two proteins. Finally, binding of the Znf cofactors to the helicases could induce changes in the conformation and function of their helicase partners. The aforementioned structural proteomic study of Prp43 found major rearrangements in the OB-fold domain on binding of the G-patch protein Ntr1 ^[45]^. G-patch and zinc-finger cofactors are structurally very different; however, both proteins appear critical in directing DEAH-RHA helicases to specific substrates and processes. A DEAH-RHA protein could employ either a G-patch or a zinc-finger cofactor. Alternatively, the same helicase could use both cofactors in the same or different processes, but no G-patch cofactor has been identified with the discussed helicases. For example, MOG-4 in nematodes (the homolog of Prp2 in yeast) could also use a G-patch protein, whereas Prp2, which binds the G-patch protein Spp2, could also have a zinc-finger cofactor. A recent structural study of the MLE (Drosophila Maleless) helicase, a RHA homolog that controls the IncRNA-mediated assembly of the ribonucleoprotein dosage compensation complex during activation of X-linked genes in Drosophila, showed a stable core with inter-domain contacts between RecA2, dsRBD2 and the OB-fold domain ^[46]^. The structure may represent a transition “on” state showing how DEAH-RHA helicases couple ATP hydrolysis and RNA translocation. Coupling of a cofactor with its helicase partner may modulate the intricate inter-domain contacts in the helicase core and thereby its functions in specific RNA or RNP recognition and remodeling.

## Future Directions

Several questions need to be addressed to better understand the mode of action of DEAH-RHA helicase•Znf protein systems in their targeted RNA processes, and their impact on diseases associated with these processes. How do Znf regulators specifically target their monomeric DEAH-RHA helicase partners? How do the helicase and Znf proteins interact and cooperate to enable specific RNA or RNP recognition and remodeling? Is the assembly of the helicase with its Znf cofactor regulated *in vivo*? Crystal structures are available for several DEAH-RHA helicases, and for ZAP, but not for any G-patch proteins nor for any DEAH-RHA helicase•Znf cofactor complexes. Structural information of a complex between a helicase with its Znf cofactor will be very important to further understand how these emerging protein partner systems function.

## Conclusions

Multi-Znf proteins are an emerging class of DEAH-RHA helicase regulators. A previous class of DEAH-RHA helicase cofactors includes G-patch proteins. Three DEAH-RHA helicase•Znf protein partnerships have been identified so far in taxonomically distant species. The recently discovered system in trypanosome RNA editing includes REH2, the largest known DEAH-RHA subfamily member, and its ^H2^F1 cofactor with eight zinc fingers. The long and independent evolutionary history of the RNA processes discussed in this short review suggests that analogous helicase•Znf systems are widespread across biology. Studies of ZAP, an antivirus protein, provide a guide to suggest potential mechanistic parallels between helicase•Znf protein systems. For example, the Znf cofactor may be a hub that brings together the RNA substrate, the RNA helicase and either exosome, spliceosome or editosome components. Also, structural studies of ZAP suggest a mechanism of RNA target recognition that may be used by other Znf cofactors. That is, a tandem array of zinc fingers creates a three-dimensional RNA-binding surface that fits complex spatial determinants in the RNA targets, not continuous sequence elements. Variations in the number and contribution of each zinc finger in the array to RNA binding may allow specific recognition of a set of related substrates. Thus, Znf proteins may directly recruit RNA substrates and thus enable modulation of local RNA structure of bound sites via helicase catalyzed unwinding. These proposed basic properties of multi-Znf cofactors would meet the needs of mitochondrial RNA editing, nuclear EJC remodeling, cytosolic viral mRNA degradation, and potentially other RNA processes involving DEAH-RHA helicases.
